# Lymphoepithelioma-Like Carcinoma of the Urinary Bladder: A Case Report

**DOI:** 10.7759/cureus.86220

**Published:** 2025-06-17

**Authors:** Nassar M Alqurashi, Thamer M Alqurashi, Wael A Hadaidi, Abdullah H Alsayed, Ibrahim Salman

**Affiliations:** 1 Urology, Al-Hada Armed Forces Hospital, Taif, SAU; 2 Urology, King Faisal Specialist Hospital and Research Centre, Jeddah, SAU

**Keywords:** bladder preserving therapy, lymphoepithelioma-like carcinoma, trans urethral resection of bladder tumor (turbt), urinary bladder ca, uro-oncology, uro pathology, urothelial bladder cancer

## Abstract

Lymphoepithelioma-like carcinoma (LELC) of the bladder is a rare variant of urothelial carcinoma that histologically resembles lymphoepithelioma of the nasopharynx. Due to its rarity, prognostic outcomes and optimal treatment strategies remain unclear.

We present the case of a 60-year-old male who presented with gross hematuria. Imaging revealed an anterior bladder wall mass. The patient underwent transurethral resection of a papillary lesion located at the bladder dome, with histopathological evaluation confirming LELC. He received neoadjuvant chemotherapy; however, follow-up cystoscopy revealed recurrence. A repeat endoscopic resection confirmed persistent disease, and the patient subsequently underwent partial cystectomy. Two follow-up cystoscopies showed no evidence of recurrence.

Bladder LELC may present in pure, predominant, or focal forms, with prognosis influenced by the proportion of lymphoepithelial components. Immunohistochemistry is essential for diagnosis. Although Epstein-Barr virus has been associated with LELC in other sites, it has not been implicated in the bladder. Multimodal treatment, typically involving surgery and cisplatin-based chemotherapy, is commonly employed. In this case, partial cystectomy led to favorable short-term outcomes. Further research is needed to establish standardized treatment protocols.

## Introduction

Lymphoepithelioma-like carcinoma (LELC) of the bladder is a rare and aggressive variant of urothelial carcinoma, first described by Zukerberg et al. in 1991, and accounts for approximately 0.4% to 1.3% of all bladder cancers, with a higher incidence in males (around 70.9%) [1]. It is most commonly diagnosed in older patients, typically presenting with painless hematuria. At diagnosis, the majority of cases are muscle-invasive, with about 87% classified as stage T2-T3 [2,3]. Histologically, these tumors resemble nasopharyngeal lymphoepitheliomas, featuring undifferentiated epithelial cells with dense lymphoid infiltration. While lymphoepitheliomas are typically associated with the nasopharynx, LELC can occur in various extra-nasopharyngeal sites, with the bladder being the most frequently affected organ in the urinary tract [1,4].

## Case presentation

A 60-year-old Saudi male presented with painless hematuria with clots that had persisted for approximately two months. His past medical history included hypertension, type 2 diabetes mellitus, benign prostatic hyperplasia, erectile dysfunction, and hypogonadism, for which he was receiving testosterone therapy. There was no significant family history of malignancy.

**Figure 1:**
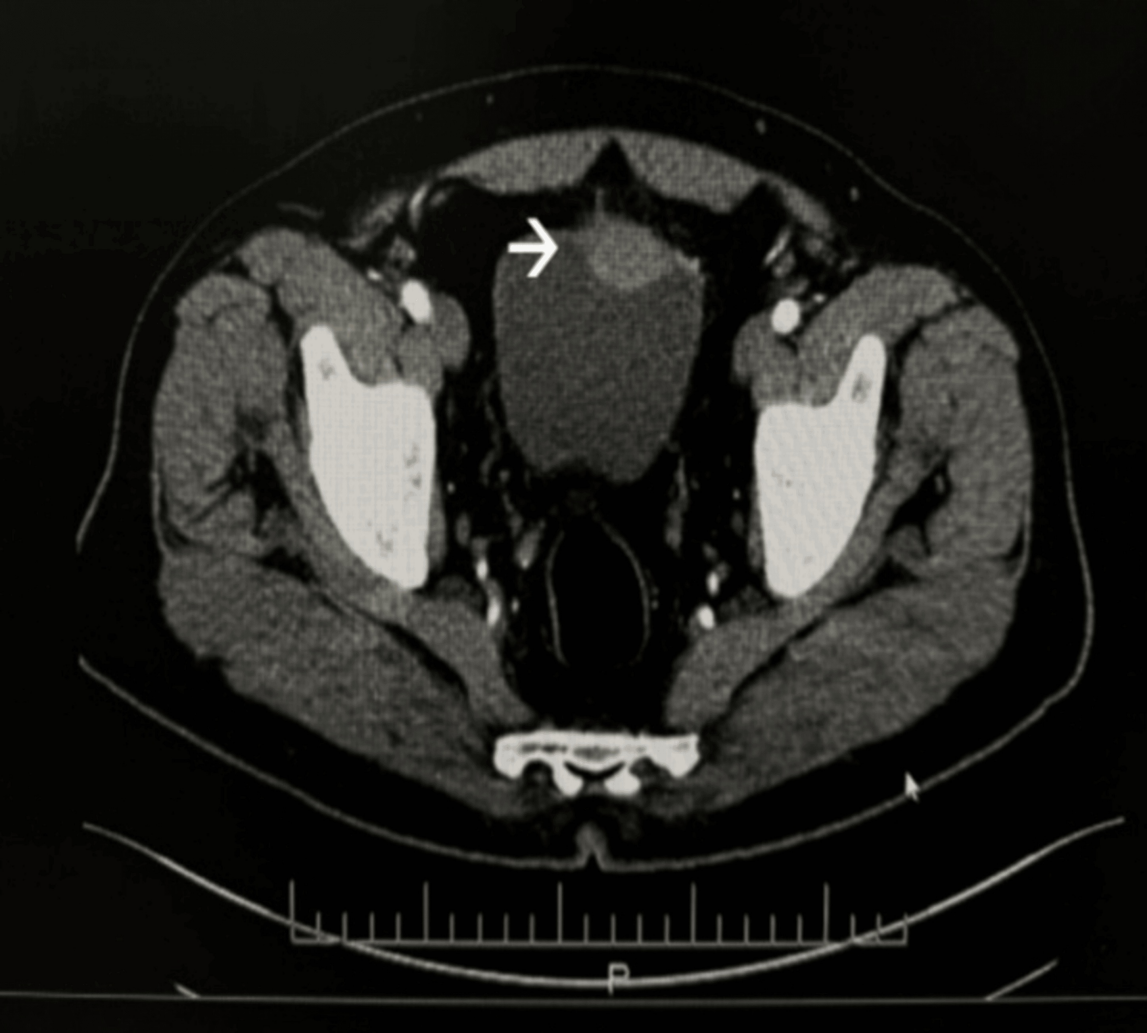
Enhanced CT showing a domal bladder mass (arrowhead).

Urine cytology was suspicious for high-grade urothelial carcinoma. A contrast-enhanced CT scan of the abdomen revealed a soft tissue mass along the anterior wall of the bladder, adjacent to the urachal remnant (Figure 1).

The patient underwent transurethral resection of the bladder tumor (TURBT). Microscopic examination revealed a neoplastic lesion composed of sheets and large nests of undifferentiated cells with dense lymphoid infiltrates, including peritumoral lymphoid aggregates and scattered lymphocytes among tumor cells. The neoplastic cells formed a syncytium, exhibiting indistinct cell borders and amphophilic cytoplasm. Nuclei were pleomorphic and vesicular with prominent nucleoli and frequent mitotic figures. Detrusor muscle invasion was noted. Based on these findings, a diagnosis of lymphoepithelioma-like variant of urothelial carcinoma was made.

**Figure 2:**
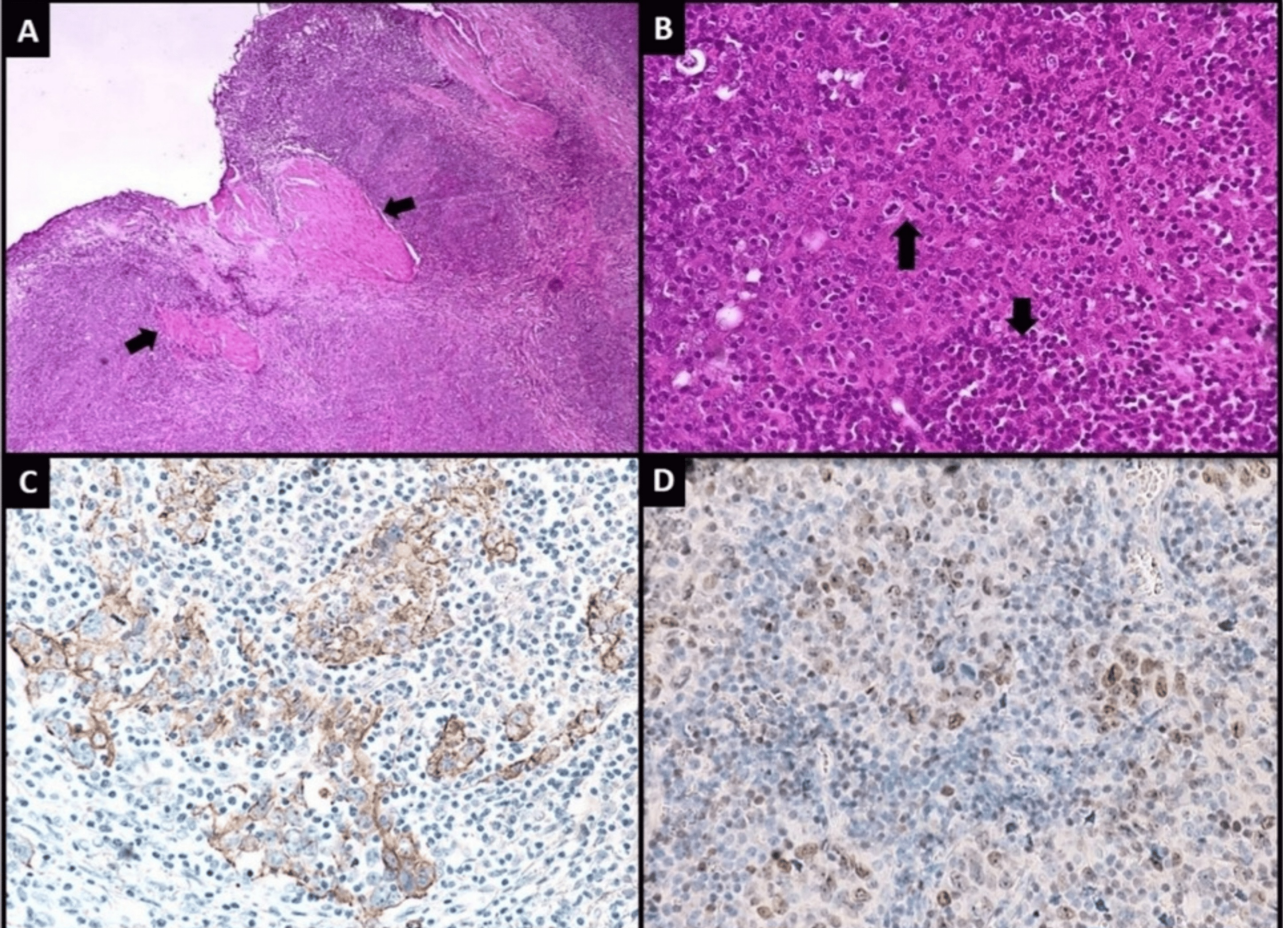
(A) Low-power view showing tumor infiltrating into detrusor muscle fibers (arrows); (B) tumor arranged in sheets and nests with dense lymphoid infiltrate (smaller arrow). Tumor cells exhibit high nuclear grade and brisk mitotic activity (larger arrow). Tumor cells show positive expression for (C) CK AE1/AE3 and (D) GATA3 on IHC staining.

Immunohistochemical staining demonstrated membranous positivity for CKAE1/AE3 and nuclear positivity for GATA3, confirming urothelial origin (Figures 2A-2D).

A follow-up cystoscopy conducted several weeks later revealed a recurrent sessile mass measuring less than 3 cm. Biopsy confirmed persistent lymphoepithelioma-like urothelial carcinoma with invasion into the muscularis propria.

The case was discussed in a multidisciplinary tumor board meeting. Neoadjuvant chemotherapy with three cycles of cisplatin and gemcitabine followed by radical cystectomy was recommended. However, the patient declined radical cystectomy and opted for bladder-preserving treatment.

He subsequently underwent partial cystectomy. Final pathology confirmed invasive urothelial carcinoma, lymphoepithelioma-like variant (rpT3). Surveillance cystoscopies performed at regular intervals during follow-up showed no evidence of recurrence, and urine cytology remained negative for malignancy.

## Discussion

Bladder LELCs are categorized based on the proportion of lymphoepithelial tissue present: pure (100%), predominant (50%-99%), and focal (<50%). These are often mixed with other types of urothelial carcinoma. The subtype significantly influences prognosis, with pure and predominant forms generally associated with better disease-free survival compared to the mixed type. Notably, mixed LELC has the highest reported mortality, followed by predominant and pure subtypes, suggesting that LELC may be less aggressive than conventional high-grade urothelial carcinoma [1,4].

While Epstein-Barr virus (EBV) has been linked to LELC in other tissues, such as the nasopharynx, EBV is not associated with bladder LELC. Instead, p53 abnormalities are believed to play a central role in the tumor’s pathogenesis. Hybridization tests for EBV-encoded RNA have consistently yielded negative results in bladder LELC cases [3].

Immunohistochemistry plays a crucial role in the differential diagnosis of bladder LELC. Tumor cells typically exhibit positivity for broad-spectrum epithelial markers such as GATA3, epithelial membrane antigen (EMA), AE1/AE3, CK7, and CK8, supporting their urothelial origin [1].

Due to the rarity of bladder LELC, there is no universally accepted treatment protocol. Various therapeutic strategies have been proposed, and many experts advocate a multimodal approach. While some support TURBT for localized disease, others consider radical cystectomy the preferred option, particularly in cases with muscle invasion. Additionally, adjuvant radiochemotherapy has been suggested following either conservative or radical surgical interventions. Nevertheless, most studies recommend the routine use of cisplatin-based chemotherapy as adjuvant therapy after TURBT or cystectomy to improve clinical outcomes [3].

## Conclusions

LELC of the bladder is a rare variant of urothelial carcinoma with distinct histopathological features and an uncertain clinical course. Due to its rarity, there is no standardized treatment that guarantees improved survival outcomes. In our case, the patient underwent partial cystectomy and showed no evidence of recurrence on follow-up cystoscopies. This suggests that bladder-preserving approaches may be feasible in selected patients. However, additional studies are needed to assess the prevalence, safety, and long-term outcomes of this treatment strategy in broader patient populations.
